# Facilitation activities for change response: a qualitative study on infection prevention and control professionals during a pandemic in Brazil

**DOI:** 10.1108/JHOM-12-2020-0506

**Published:** 2021-08-23

**Authors:** Luís Irgang, Magnus Holmén, Fábio Gama, Petra Svedberg

**Affiliations:** Department of Innovation Management, Halmstad University , Halmstad, Sweden; Department of Business Administration, FURB , Blumenau, Brazil; Department of Public Administration, UDESC , Florianopolis, Brazil; Department of Health and Care, Halmstad University , Halmstad, Sweden

**Keywords:** Facilitation activities, Change response, Implementation of changes, Evidence-based interventions, COVID-19 pandemic, Infection prevention and control professionals

## Abstract

**Purpose:**

Facilitation activities support implementation of evidence-based interventions within healthcare organizations. Few studies have attempted to understand how facilitation activities are performed to promote the uptake of evidence-based interventions in hospitals from resource-poor countries during crises such as pandemics. This paper aims to explore facilitation activities by infection prevention and control (IPC) professionals in 16 hospitals from 9 states in Brazil during the COVID-19 pandemic.

**Design/methodology/approach:**

Primary and secondary data were collected between March and December 2020. Semi-structured interviews were conducted with 21 IPC professionals in Brazilian hospitals during the COVID-19 pandemic. Public and internal documents were used for data triangulation. The data were analyzed through thematic analysis technique.

**Findings:**

Building on the change response theory, this study explores the facilitation activities from the cognitive, behavioral and affective aspects. The facilitation activities are grouped in three overarching dimensions: (1) creating and sustaining legitimacy to continuous and rapid changes, (2) fostering capabilities for continuous changes and (3) accelerating individual commitment.

**Practical implications:**

During crises such as pandemics, facilitation activities by IPC professionals need to embrace all the cognitive, behavioral and affective aspects to stimulate positive attitudes of frontline workers toward continuous and urgent changes.

**Originality/value:**

This study provides unique and timely empirical evidence on the facilitation activities that support the implementation of evidence-based interventions by IPC professionals during crises in hospitals in a resource-poor country.

## Background

Healthcare professionals call for novel approaches to assist in implementing evidence-based interventions in resource-poor countries ( Yapa and Bärnighausen, 2018 ). Facilitation activities within resource-poor settings are highly complex and uncertain. The difficulties stem from restrictions in responding to contextual barriers given the limited human, financial and structural resources ( Costa *et al.* , 2017 ). This is an enduring issue during crises such as pandemics, where conflicting guidelines, overwhelming information and limited resources hinder rapid implementation of evidence-based interventions ( Rebmann, 2010 ; Moon *et al.* , 2015 ).

Facilitation activities refer to techniques designed to enable individuals and teams to work collaboratively on agreed areas for improvement, creation and sustaining changes in healthcare provision ( Moussa *et al.* , 2019 ). Unlike other activities, facilitation activities focus on enabling and influencing workplace culture, upskilling and empowering team members to streamline changes ( Young *et al.* , 2019 ; Cranley *et al.* , 2017 ; Harvey and Lynch, 2017 ; Lessard *et al.* , 2016 ). Facilitation activities are crucial during pandemics, as frontline workers tend to fail in adhering to changes in such circumstances. For example, studies show that frontline workers often struggle in engaging in evidence-based interventions during pandemics, mainly due to the increased workload, physical and psychological stress, fear and distrust of new interventions ( McMullan *et al.* , 2016 ; Rutkow *et al.* , 2017 ). Fortunately, facilitation activities can overcome barriers and motivate positive attitudes toward changes ( Houghton *et al.* , 2020 ). Yet, the study of implementation during crises is a relatively young field of investigation, and little is known about performing facilitation activities to promote the uptake of new and adapted evidence-based interventions introduced during crises such as pandemics.

The success of implementation strategies depends largely on the use of effective and context-specific facilitation activities that fit the circumstances ( Harvey and Lynch, 2017 ; Kirchner *et al.* , 2020 ). Notably, prior studies have mainly examined facilitation activities in developed economies and under non-crisis contexts (e.g. Young *et al.* , 2019 ; Ritchie *et al.* , 2020 ) (see Table 1 ). While these studies make valuable contributions, they do not examine deeply how the context constrains the use of facilitation activities during crises where rapid adherence to new evidence-based practice is needed. Moreover, the literature on facilitation activities often emphasizes organizational and structural features rather than the relation between facilitators and frontline workers (e.g. Nguyen *et al.* , 2020 ). A recent literature review about the experience of nurses, doctors and other healthcare professionals indicates that low confidence in evidence, uncertainties about national or internal hospital guidelines, conflicting individual beliefs (e.g. fear) and work overload represents major barriers ( Houghton *et al.* , 2020 ). Despite a wide range of facilitation activities, few implementation studies report sufficient detail on the interplay between facilitators, facilitation activities and recipients' responses to change.

Recent studies in implementation science and organizational change, however, indicate *change response* as a useful theoretical lens to understand the capacity of individuals to change ( Nilsen *et al.* , 2019 ; van den Heuvel *et al.* , 2017 ). Change response literature stresses that recipients' responses to change depends on the combination of cognitive, behavioral and affective understanding and stresses that in the general case, it is insufficient for facilitation to address only one of these aspects ( Bouckenooghe, 2010 ). More precisely, change response is conceptualized as a tri-dimensional attitude composed of three components: cognitive (opinions about changes, i.e. advantages or disadvantages, usefulness and necessity), behavioral (actions for or against changes) and affective (feelings and emotions about changes) ( Elizur and Guttman, 1976 ). These cognitive, behavioral and affective aspects encompass both positive and negative recipients' responses about changes ( Bouckenooghe, 2010 ).

To date, the interplay between facilitation activities and change recipients have not been well described, especially how facilitation activities are operationalized by facilitators in complex implementation of new and adapted evidence-based interventions during crises ( Houghton *et al.* , 2020 ). This is particularly relevant in the context of infection prevention and control (IPC) during pandemics, as changes might be continuous, frequent, contradictory and recurring ( Rebmann, 2010 ). Here, the IPC professionals (mostly nurses and infectious diseases physicians with academic training in the field of IPC) are challenged with promoting, disseminating and implementing IPC policies in response to multiple recommendations from health organizations and experts ( Billings *et al.* , 2019 ; World Health Organization, 2020a ). While regular IPC professionals' duties include, among of other things, leadership support, surveillance of infectious disease processes and application of standard and transmission-based precautions ( Centers for Disease Control and Prevention, 2017 ), in crises contexts (e.g. pandemics) IPC professionals are challenged with the need of implementing continuous changes in IPC routines. This includes, for example, the adoption of alternative training methods, adapting hospital facilities and changing workflows and patient care routines ( Wong *et al.* , 2017 ).

Against this backdrop, this study aims to explore the facilitation activities by IPC professionals during crises in hospitals in a resource-poor country. To advance our understanding of facilitation activities during crises, we adopted the theory of change response as the conceptual and theoretical framework to explore the facilitating activities developed by IPC professionals enrolled as facilitators in implementing evidence-based interventions during the COVID-19 pandemic in Brazil.

## Method

### Study design

To explore facilitation activities by IPC professionals during the COVID-19 pandemic in hospitals in Brazil, we adopted a qualitative descriptive study design ( Sandelowski, 2000 ). This naturalistic inquiry approach is suitable for two reasons. First, an advanced understanding on the facilitation activities for implementation of evidence-based interventions relies on exploring the roles and perceptions of facilitators, who perform facilitation activities and engage people in the change process ( Young *et al.* , 2019 ). Therefore, the approach enables rich descriptions on individual and group experiences when performing facilitation activities ( Mansfield *et al.* , 2018 ). Second, pandemics represent a complex and uncertain circumstance, as they stress deficiencies in IPC practices and require rapid change response from healthcare professionals ( Maves *et al.* , 2019 ). Therefore, a qualitative approach is valuable to explore how crisis contexts may drive individuals' attitudes toward or against changes ( Lam *et al.* , 2019 ).

### Study setting

The study focused on IPC professionals working in 16 hospitals from 9 states in Brazil and distributed over 4 regions: 2 hospitals are in the south, 4 hospitals in the southeast, 2 hospitals in the northeast and 1 hospital in the north. All hospitals are in urban areas and provide health services to populations from regions ranging from 1 to 12 million inhabitants. Among them, seven hospitals are public and nine are private (of which seven do business with the public health system). The hospitals have, on average, 360 hospital beds and 2,600 employees. Besides, all hospitals have dedicated and experienced IPC professionals and have implemented new and adapted evidence-based practices during the COVID-19 pandemic.

We choose Brazilian hospitals as they represent critical research settings in a resource-poor country ( Palinkas *et al.* , 2015 ), due to four reasons. First, Brazil is a middle-income country, and hospitals in such countries often have limited financial and technical resources, which represents a more challenging context in the implementation of new and adapted evidence-based interventions during crises ( Bedford *et al.* , 2020 ). Second, there were several cases of corruption involving fraud, overbilling and embezzlement of financial resources in the purchase of hospital equipment and supplies during the COVID-19 pandemic ( Mello and Grillo, 2020 ). This led to a shortage of basic products in hospitals and some evidence-based interventions needed to be adapted, since some IPC practices rely on the availability of suitable materials. Third, the institutional conflicts and the lack of coordination between the Federal Government and the state governments hampered the consolidation of a national strategic plan to guide the hospital IPC activities ( Ricard and Medeiros, 2020 ). As a result, hospitals received conflicting recommendations on the evidence-based interventions that should be implemented. Fourth, myths or misleading information (called “ *fake news* ” in this study) through social media led people to question the severity of the pandemic and to discredit the IPC recommendations ( de Sousa Júnior *et al.* , 2020 ), which affected the credibility of IPC professionals when performing facilitation activities for implementation of evidence-based interventions.

### Sampling and data collection

The data collection took place from March to December 2020, primarily based on exploratory, semi-structured and follow-up interviews with nurses and physicians enrolled as IPC professionals in Brazilian hospitals during the COVID-19 pandemic. To recruit respondents, we combined two purposeful sampling strategies: criterion and snowball sampling ( Palinkas *et al.* , 2015 ). First and foremost, the respondents must have the requisite knowledge and experience regarding the phenomenon being researched ( Bradshaw *et al.* , 2017 ). Therefore, for participation criteria we defined respondents as follows: being enrolled as a senior IPC professional ( World Health Organization, 2020a ) in a hospital in Brazil, being engaged in facilitation activities during the COVID-19 pandemic and having experience with implementation of evidence-based interventions in IPC for at least three years. We recruited 16 respondents through invitations posted on online discussion forums about IPC and on social media (LinkedIn® and Facebook®). During the interviews, respondents were asked to recommend other IPC professionals as potential respondents. From these recommendations, we recruited five more respondents.

The interviews were conducted in three stages. First, exploratory interviews were conducted with two healthcare professionals with expertise in nursing and public health, to gather insights on facilitation activities for implementation of evidence-based interventions during a pandemic. After the exploratory interviews and based on extensive review on facilitation literature, we elaborated a semi-structured interview protocol containing 15 questions. The questions addressed, for example, how IPC professionals dealt with multiple and continuous changes issued by health authorities and experts, and based on that, how they organized and performed the facilitation activities to drive and motivate frontline workers' attitudes toward the changes. Second, we conducted individual interviews with 21 IPC professionals ( *n* = 21). Data saturation was reached after 16 interviews, stage which new categories did not seem to be forthcoming and new data seemed redundant ( Guest *et al.* , 2006 ). Third, follow-up interviews were conducted with two respondents in order to validate and legitimize the data. The interviews were conducted by phone and in Portuguese by the first author, and then recorded, transcribed verbatim and translated to English. Table 2 provides descriptive information of interviews and respondents' characteristics.

To triangulate the data, we accessed secondary data and documents. We considered documents, guidelines and healthcare experts and authorities' technical notes related to changes on IPC practices issued from March 11, 2020, when the World Health Organization declared the COVID-19 outbreak a global pandemic ( World Health Organization, 2020b ). Furthermore, respondents provided hospital documents such as crisis containment plans and technical protocols on IPC practices. These documents were used to support data description and interpretation.

### Data analysis

We used a thematic analysis technique ( Braun and Clarke, 2006 ) as it allows to identify and classify patterns in the data by combining theory-driven and data-driven approaches. The data analysis followed five overlapping steps: familiarization with the data, generating initial codes, constructing themes, revising and defining themes.

For *familiarization with the data* , the first author transcribed, translated from Portuguese to English, and then summarized the interviews transcriptions and the additional documents. Later, the transcriptions of the interviews and additional documents were shared with all the authors. As the data analysis took place during the pandemic, all the authors engaged in discussions about the research phenomenon and shared additional materials such as newspaper articles and guidelines.

In *generating initial codes* , the authors identified patterns throughout the dataset and clustered latent codes around similar meanings from an inductive approach ( Fereday and Muir-Cochrane, 2006 ). Later, the authors developed a code manual with definitions of what the first-order codes concern and how to know when they occur throughout the dataset.

In *constructing themes* , the authors adopted a hybrid perspective of inductive and deductive approaches ( Fereday and Muir-Cochrane, 2006 ) to formulate a list of second-order themes to label the clustered first-order codes. The second-order themes are interpretive concepts that connect the change response theory and the empirical data.

In *revising themes,* the authors delineated the second-order theme's boundaries to clarify the essence and scope of each theme. The themes were checked against the whole dataset and fitted the initial second-order themes according to their power to aggregate the first-order codes and reflect their content. The themes were then classified according to the three aspects suggested by the change response theory (cognitive, behavioral and affective) ( Elizur and Guttman, 1976 ).

**Figure 1:**
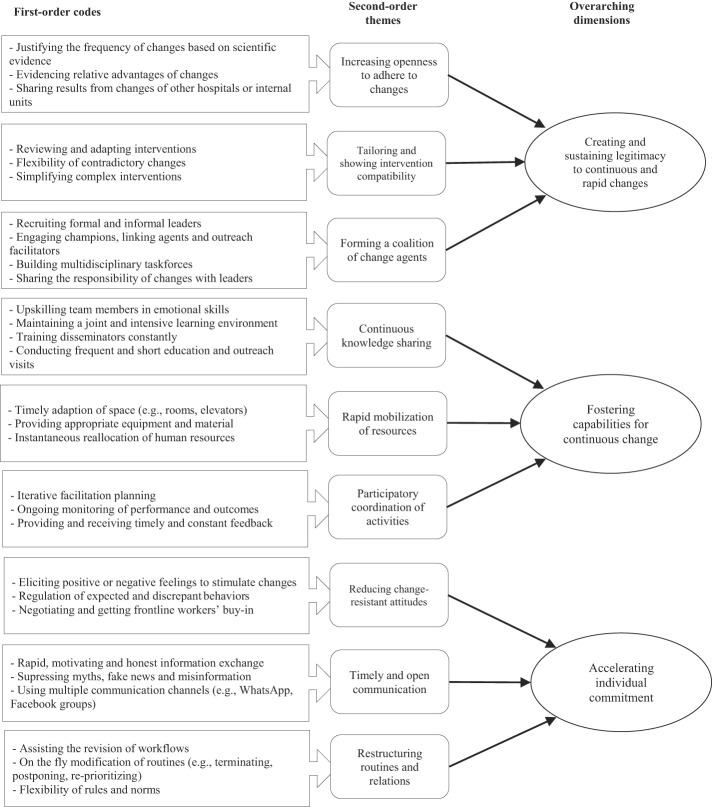
Data structure of the findings

In *defining themes* , the second-order themes were re-examined and distilled into three overarching dimensions related to the theory of change response. Later, we conducted follow-up interviews with two respondents in order to ensure the confirmability and dependability of the findings ( Lincoln and Guba, 1985 ). Figure 1 illustrates the data structure where the interplay between first-order codes, second-order themes and overarching dimensions is presented. The configuration of the data structure resulted from extensive analysis and 17 discussion rounds among the authors of the study.

## Findings

The facilitation activities performed by IPC professionals during the COVID-19 pandemic are described in three overarching dimensions: (1) *creating and sustaining legitimacy to continuous and rapid changes* , (2) *fostering capabilities for continuous change* and (3) *accelerating individual commitment* . The following sub-sections present the three dimensions and by the end how they are related to cognitive, behavioral and affective aspects. Quotations from the semi-structured interviews are provided to support the findings.

### Creating and sustaining legitimacy to continuous and rapid changes

This dimension refers to the promotion of long-term acceptance of new and adapted evidence-based interventions. A sustained legitimacy is achieved when there is a high degree of congruence on how the frontline workers perceive the benefits and necessity for the changes being continuously implemented. This dimension aggregates three second-order themes: *increasing the openness to adhere to changes* , *tailoring and showing intervention compatibility* and *forming a coalition of change agents.*

*Increasing the openness to adhere to changes* encompasses facilitation activities that are related to stimulate the uptake of new and adapted evidence-based interventions by discussing with frontline workers the benefits and the need for changes, or presenting negative consequences of struggling or rejecting the changes. IPC professionals increase the credibility and demonstrate the need for changes by presenting reliable information sources such as scientific evidence and experts and authorities' recommendations. An IPC coordinator remarked:

When we explain why we are changing, we always emphasize [that] this is in accordance with the knowledge we have today. And tomorrow, we may have to change again and again and again. But everyone needs to understand that we are following the science.

Even though new changes may be frequent and unpredictable, IPC professionals share implementation results from other hospitals and internal units to reinforce that evidence-based interventions are standardized and must be implemented in all settings. Moreover, selected cases of successful and failed implementation are presented as they stimulate positive opinions toward the interventions and contribute to the legitimacy of the changes. One nurse said:

I think our best strategy was to show positive and negative results from other hospitals. When some new procedure was about to be implemented, we first tried to illustrate with real examples what could happen if we did not follow the recommendations.

*Tailoring and showing intervention compatibility* comprises facilitation activities that aim to adapt the interventions to the contextual factors. According to the respondents, some evidence-based interventions are unfeasible or useless because they do not fit with the structure or context of the hospitals, regardless the size, location or ownership of the hospital. In sum, this happens because health authorities issue generic guidelines and recommendations, which often do not correspond to the hospitals' needs or are out of alignment with internal policies or resources. Therefore, IPC professionals review and adapt interventions before implementing them within frontline workers. Yet, some interventions are too complex to implement (e.g. demanding the engagement of multiple actors) or too disruptive and contradictory in comparison with existing IPC practices. In such scenarios, IPC professionals promote the flexible or adapted interventions, which include the partial implementation acceptance, as one nurse detailed:

The standard lifespan of an N95 mask usually practiced in our hospital was five days. One of ANVISA's [Brazilian Health Regulatory Agency] recommendations was to extend it to 30 days. The difference was enormous, and we could not implement it here. They [the frontline professionals] did not accept it [the change on masks' lifespan]. Then, we agreed to adopt a lifespan of 15 days.

*Forming a coalition of change agents* encompasses facilitation activities that engage opinion leaders who will motivate frontline workers' positive opinions toward the interventions being implemented. Change agents may have a formal leadership role (e.g. top and middle managers and frontline supervisors) or informal leadership role (e.g. champions, linking nurses and experienced professionals). Engaging top managers (also known as executive engagement) across different departments in the implementation process generates a sense of shared responsibility for the successful implementation and legitimizes the changes that need to be accepted and sustained over time. Engaging informal leaders is also relevant, as these actors can persuade frontline workers to accept changes. An IPC coordinator noted:

At the beginning of the pandemic, the [hospital] directors joined us in some trainings in specific units. We noticed that the resistance against changes decreased in those units. Then we started to include the participation of at least one [of the directors], especially during critical implementations.

Respondents emphasized the importance of having previously identified agents of change, who had already been engaged in implementation processes before. An IPC coordinator stated:

We had mapped [ *sic* ] influencers long before the pandemic. This helped us because we know that these leaders are enthusiastic about changes and make the implementation better [if they guide] groups that are more resistant [to changes].

Overall, the dimension creating and sustaining legitimacy to continuous and rapid changes consist mainly of cognitive-oriented activities. Such activities focus on increasing the opinions about change, their usefulness and benefits. Frontline workers were encouraged to believe the continuous changes seem doable and thereby being opened to try out the changes. This dimension stresses the legitimacy of changes in contexts where ongoing changes need to be perceived as necessary to be rapidly implemented and occasionally institutionalized in the long-term.

### Fostering capabilities for continuous change

This dimension refers to providing frontline workers with the skills, tools and guidance needed to implement changes. This dimension aggregates three second-order themes: *continuous knowledge sharing* , *rapid mobilization of resources* and *participatory coordination of activities.*

*Continuous knowledge sharing* comprises facilitation activities that stimulate a permanent and collaborative teaching and learning environment. While the changes were frequent and need to be rapidly implemented, the traditional ways of knowledge sharing (e.g. periodic educational meetings and intensive training programs) are inefficient. Instead, IPC professionals adopt short training sections and meetings, which are informal and targeted according to the needs of each hospital unit. The traditional learning model was replaced by a collaborative format, where professionals share knowledge in an active and informal way and receive timely updates through short outreach visits. Perhaps most importantly, in addition to the shared technical knowledge, IPC professionals started sharing relaxing and concentration techniques to decrease the stress and stimulating positive psychological conditions to the acceptance of continuous changes. An IPC consultant nurse detailed:

Before transmitting the technical part, we teach relaxation and concentration exercises. This has helped professionals to keep emotional control, to reduce stress and thereby learn more quickly how to execute practices correctly. If we just go there and start asking to change again and again and again, without any emotional support, they will freak out.

In line with the formation of a coalition of change agents, IPC professionals recruit and prioritize training to disseminators, also known as *boundary spanners* (see Pronovost *et al.* , 2017 ), which are key professionals with formal and informal roles of sharing knowledge within their hospital units. Such professionals engage department colleagues into a collaborative learning process and support them in developing technical and emotional competences to facilitate the uptake of continuous changes. An IPC coordinator commented:

In each (hospital) unit there is a kind of an ‘angel’, who is a professional who is easy to learn and teach. This professional receives updates firsthand and then disseminates this knowledge over the unit.

*Rapid mobilization of resources* includes the facilitation activities that aim to make physical, technological and human resources available to frontline workers in a timely and appropriate manner. While changes during pandemics are continuous, frequent and might be urgent, frontline workers must have rapid access to appropriate resources as a requirement to properly carry out the interventions. This is problematic for hospitals, regardless their size or ownership, especially when the mobilization of resources is aimed to promote structural changes.

Although this might not be a main prerogative of IPC professionals, they support and advise managers on the needs and the distribution of resources. One reason is that hospital management discuss with IPC professionals about the needs of resources from an overarching and evidence-based perspective. In addition, the IPC professionals are uniquely qualified to prioritize and manage of resources from an IPC perspective. An infectious disease physician remarked:

As soon as a new recommendation is issued, the first thing we do is check with the supply department if the availability and amount of material we need for the intervention. In case they do not have enough materials, we try to make rearrangements between the departments, to make sure the professionals have the appropriate material on time.

Additionally, respondents reported two main challenges in reallocating human resources. First, there is an increased demand for hospital services due to COVID-19. Second, respondents also perceived high rates of absenteeism and turnover during the pandemic. Therefore, recruitment of professionals and rearrangement of workforce over hospital units are needed. To address these issues, IPC professionals collaborated with human resources (referred to as human development units) to prioritize units (and activities) in the hospital where it would be necessary to increase the workforce. In addition, IPC professionals contribute in defining criteria for the re-deployment over hospital units, with the aim of meeting the implementation tasks without compromising the level and quality of services.

*Participatory coordination of facilitation activities* presents the active and iterative processes of managing the facilitation activities that aim to make the evidence-based interventions easier to accept. Respondents reported that, due to the frequency of changes, facilitation activities must be monitored in real time and adapted according to prompt and constant feedback from both frontline workers and managers, which ultimately empowers the frontline workers and fosters group cohesion. Additionally, the planning of facilitation activities must be ongoing and adaptive, as an infectious disease physician pointed out:

Sometimes the order of the day is: ‘let's do it first and wait for the results’. Later, we do plan based on the results and with the participation of multiple leaders and from a multi-disciplinary perspective (…). This may sound weird, but if we are in a hurry, planning is not a priority.

Overall, fostering capabilities for continuous change encompass facilitation activities that are predominantly behavior-oriented, as such activities drive positive intentions toward the changes. This is about the readiness and acceptance for change in the sense of highlighting the need of primary competences, resources and guidance to promote positive reactions and the rapid uptake of continuous changes. This dimension underscores the perspective of timely adaptation and reallocation of resources to reduce the objections, protests and complaints of change by frontline workers.

### Accelerating individual commitment

This dimension refers to stimulating frontline workers to develop positive emotions and sympathy toward continuous changes. As some evidence-based interventions are urgent and need to be rapidly integrated, frontline workers must be willing to accept continuous changes and committed to cooperate with the rapid implementation. This dimension aggregates the second-order themes as follows: *reducing change-resistant attitudes, timely and open communication* and *restructuring routines and relations.*

*Reducing change-resistant attitudes* refers to the facilitation activities that aim to mitigate opposition against the changes. Respondents detailed that continuous changes caused an increase in the workload and raised the stress level of frontline workers. First, some interventions are too complex, and frontline workers feel skeptical about the trustworthiness and credibility of such interventions. Second, some interventions are perceived as contradictory or ambiguous, because they are not in accordance with the protocols normally followed by professionals, or because the interventions reintroduced previously discarded concepts or techniques. This generated change-resistant attitudes, which need to be continually mitigated through formal behavior regulation measures or by negotiating and getting buy-in. An IPC nurse said:

We tried to motivate them [the frontline workers] to accept changes spontaneously. But some prefer to remain in their comfort zones, so we had to introduce ground rules to ensure that everyone adheres to what needs to be implemented.

Furthermore, respondents argued that positive and negative emotions can also be stimulated in order to motivate frontline workers to accept the changes, as an IPC coordinator complemented:

We clarify that if they [the frontline workers] follow the rules, they will be safe, as well as ensuring the safety of patients. On the other hand, if they do not follow the rules, they are at risk of being infected, and can transmit the virus to their own family members … It is scary, but this is the reality.

*Timely and open communication* refers to the facilitation activities that aim to ensure the correct and on-time information about the changes. As the changes are urgent and need to be rapidly implemented most of the time, IPC professionals adopted non-standardized methods of communication. This allowed for a faster, clear and, although informal, more effective exchange of information. An IPC nurse pointed out:

We never thought that talking about evidence-based interventions through WhatsApp groups could be so successful ( …). Communication is fast and open (…) and it seems that colleagues also approve, because I see them reading our WhatsApp messages with enthusiasm.

Furthermore, alternative communication channels not only approximate IPC professionals and frontline workers, but also help IPC professionals to suppress and correct myths and “ *fake news* ” about the changes, as one infectious disease physician noted:

By WhatsApp, we can answer questions from employees [frontline workers] and patients. And it is very useful, because when they receive “ *fake news* ”, they forward the messages to us, and we check and immediately contest.

*Restructuring routines and relations* refers to facilitation activities that promote changes in organizational processes. This includes rethinking the organizational architecture, norms and rules and redesigning the internal workflows. For example, some changes are only possible to be implemented after the suspension of current practices or the complete replacement of significant processes for the normal functioning of the organization, as a nurse stated:

We stopped elective procedures to give preference to the treatment of COVID-19 (…). We then redesigned the process flows to facilitate the new routines of patient care.

Furthermore, IPC professionals support managers to adapt internal rules in order to make frontline workers more flexible and aware of changes. One IPC nurse pointed out:

Before, it was forbidden to have lunch inside the units. Now we can. Before, each employee had a permanent break time. Now it is according to the demand of patients. Before it was forbidden to work from home. Now it is recommended. There have been lots of big changes in our organizational dogmas.

Overall, accelerating individual commitment encompasses primarily affective-oriented facilitation activities. In line with the notion of commitment for change, our findings show that IPC professionals stimulate positive emotions toward the changes in the sense of creating distributed leadership, i.e. a responsibility to contribute to the successful implementation of changes (see Willis *et al.* , 2016 ). However, considering that changes during the pandemic are unpredictable and need to be rapidly implemented, IPC professionals perform facilitation activities to accelerate the commitment of frontline workers toward the changes, especially by motivating the engagement in the implementation process through efficient and rapid communication systems.

## Discussion

The findings show that the facilitation activities performed by IPC professionals during the COVID-19 pandemic focused on creating and sustaining legitimacy to continuous and rapid changes, fostering capabilities for continuous changes and accelerating individual commitment. In line with Houghton *et al.* (2020) , our findings showed that during crises, frontline workers are working outside their comfort zones where they have to provide timely responses. This situation leads them to become insecure, anxious and fatigued. To handle this situation, the IPC professionals make use of facilitation activities targeting the three cognitive, behavioral and affective aspects to alleviate the imposed pressure.

Our findings indicate that there are three key set of facilitation activities performed by IPC professionals during pandemics: forming a coalition of change agents, rapid mobilization of resources and timely and open communication. First, in line with previous studies (e.g. Ritchie *et al.* , 2020 ; Lessard *et al.* , 2016 ), our study highlights the importance of engaging change agents to facilitate the acceptance of changes among frontline workers. The change agents have a dual role: first and foremost, as formal or informal leaders they can persuade other professionals regarding the benefits and the need to accept new or adapted interventions, even if such interventions are frequent and often contradictory. Furthermore, the change agents also serve as boundary spanners ( Pronovost *et al.* , 2017 ), as they receive and disseminate knowledge about new and adapted interventions within the hospital units more efficiently than the IPC professionals. The change agents often have free access to other teams of professionals and are perceived as credible and trustworthy. Therefore, in line with Young *et al.* (2019) , IPC professionals rely on the credibility and trustworthiness of change agents to promote and support the acceptance of new and adapted evidence-based interventions among healthcare professionals.

Second, our study suggests that rapid mobilization of resources to enable the implementation of changes in IPC practices is critical during a pandemic crisis. In line with Moussa *et al.* (2019) , our findings show that rapid mobilization of resources is problematic for any facilitation activity regardless of the size, ownership or location of the hospital. However, it becomes particularly pronounced because of the combination of the need to implement rapid and unexpected changes and the imminence of investing or reallocating scarce resources that imply in additional costs to the hospital or demand structural changes that take long time to be placed.

The rapid mobilization of resources occurs in two ways. In the short-term there are a frequent number of changes in the hospital structure which may generate chain reactions, implying continuous and recurring changes. Examples include the adoption and expansion of hospital units dedicated to treat COVID-19 patients, the implementation of testing sites for healthcare professionals, patients and visitors and the installation of physical barriers to prevent intra-hospital contamination. In the long-term, lasting weeks and months, the changes may not disappear because of the highly volatile context of a pandemic crisis. The volatile environment, with new rules, expectations, stresses the IPC professionals and hospital staff leading to a great scarcity of attention, time and physical resources during the crises. This implies professional roles and routines may temporarily break down, as they are unable to timely deal with the greatly increased number of novel decisions and adjustments of work practices during crises. Rapid mobilization of resources is, therefore, an ever-present issue during crises that hinder rapid implementation of evidence-based interventions ( Rebmann, 2010 ; Moon *et al.* , 2015 ).

Third, our findings demonstrate that, during a pandemic crisis, timely and open communication is essential to ensure that the healthcare professionals, patients and patients' relatives receive timely and reliable information. Prior studies indicated that, during health crises, communication issues often hinder the implementation of evidence-based interventions, especially due to conflicting and overwhelming information ( Rebmann, 2010 ; Moon *et al.* , 2015 ). This was especially problematic during the COVID-19 pandemic in Brazil, as myths or misleading information (so called “ *fake news* ”) spread on social media led people to discredit healthcare organizations and experts and, therefore, imposed barriers for the acceptance of IPC recommendations ( de Sousa Júnior *et al.* , 2020 ), even among healthcare professionals. To mitigate this issue, IPC professionals intensified communication with professionals, patients, family members and the community in general and adopted alternative and channels to share updates, disseminate recommendations and suppress *fake news* .

## Conclusion and implications

In this study, we explored how facilitation activities were developed and performed by IPC professionals to promote the uptake of new and adapted evidence-based interventions in Brazilian hospitals during the COVID-19 pandemic. The facilitation activities aimed to increase the frontline workers' perceptions of usefulness, necessity and advantages of the changes, generate positive emotions and satisfaction toward the changes and provide mechanisms for rapid acceptance of changes. We found change-response framework ( Elizur and Guttman, 1976 ) to be useful for a nuanced understanding of how IPC professionals use facilitation activities during crises in hospitals in a resource-poor country. We identified three main sets of facilitation activities to motivate positive change responses: (1) *creating and sustaining legitimacy to continuous and rapid changes* , (2) *fostering capabilities for continuous change* and (3) *accelerating individual commitment* . Each dimension aggregates three second-order themes that are related to cognitive, behavioral and affective aspects.

Our study highlighted three main sets of facilitation activities undertaken by IPC professionals: *forming a coalition of change agents, rapid mobilization of resources* and *timely and open communication* . Forming a coalition of change agents embrace recruiting formal and informal leaders, engaging champions, linking agents and outreach facilitators, building multi-disciplinary taskforces and sharing the responsibility of changes with leaders. Rapid mobilization of resources includes timely adaption of space, providing appropriate equipment and material and instantaneous reallocation of human resources. Timely and open communication comprises rapid, motivating and honest information exchange and suppressing myths, fake news, misinformation and using multiple communication channels.

This study contributes to the implementation literature and provides novel knowledge for practical use in implementation of changes in healthcare settings. First, in line with the change response literature, we showed that facilitation activities embrace each of the cognitive, behavioral and affective aspects. While specific facilitation activities may be more heavily oriented toward one aspect, during crises others are more generic and occasionally overlapped with all three aspects. The reason for this is the importance of rapid, frequent and recurrent responses during crises where frontline workers are working outside their comfort zones leading to feelings of insecurity, anxiety and fatigue. To handle this, facilitation act on all three aspects, while under “normal” circumstances in healthcare, fewer activities may e.g. involve the affective aspect, given that the work is within the normal roles.

Second, this study advances the implementation literature by responding to the call about how healthcare professionals in resource-poor countries develop novel approaches to overcome resources constraints ( Yapa and Barnighausen, 2018 ). Prior literature has explored and suggested critical facilitation activities to implement new and adapted evidence-based practices successfully but have underestimated contextual factors, assuming that healthcare organizations do not suffer from severe resource constraints contexts. However, the literature does not show how evidence-based practices are developed in contexts characterized by severe resource and time scarcity. This is problematic as our study showed that one of the biggest challenges in this healthcare setting during the pandemic was the management of both organizational and human resources. While changes during pandemics are continuous and urgent, rather than planned, a strategy for rapid mobilization of resources was crucial to ensure frontline workers' adherence to new interventions and to secure quality of care for patients. In order to do that, IPC professionals needed to adopt a participatory and iterative coordination of facilitation activities with both the frontline workers and managers. Therefore, the present study adds to the previous implementation literature a set of new facilitation activities that consider local constraints within resource-poor healthcare settings.

A suggested generalization of our findings is that change responses during crisis are continuous and specific. The changes are continuous in the sense that actions need to be invented to fit the specific context of a ward or a hospital, repeated, terminated, adapted and adopted and sometimes reversed altogether. This implies that facilitation activities during crisis need to be not only performed on the fly, be planned, but also re-enacted during long periods of time.

### Limitations and future research

Strength of this study is that the findings are grounded in the narratives of IPC professionals with diverse experiences of implementation of new and adapted evidence-based interventions during pandemics, recruited from 16 hospitals from 9 states in Brazil. The credibility of the study was strengthened through the diverse sample, the rich data from both exploratory interviews and follow-up interviews and by triangulating the interview data with secondary data and documents. When considering the findings, it is essential to note that the findings reflect the experiences from the included participants, and this could limit the generalizability of findings. However, to our knowledge there are not any qualitative studies that explore IPC professionals' perspectives on facilitation activities in relation to the implementation of new and adapted evidence-based interventions during pandemics in hospitals from resource-poor countries. Thus, the findings offer valuable insights and may inform practice in how to address facilitation challenges and plan for implementation strategies during crisis in hospital from resource-poor countries. Further studies should include individuals from top management, policy positions and frontline workers, in order to reach a more comprehensive understanding of the implementation of evidence-based interventions during crisis in resource-poor healthcare settings. Further studies should also include larger and more representative samples of respondents, aiming to confirm or reject our findings by employing quantitative methods. Accordingly, it will be necessary for future studies to investigate if and how organizational aspects (e.g. size, ownership and location of the hospitals), professional aspects (e.g. academic and clinical training, years of experience with implementation of evidence-based practices) and the nature of the changes (e.g. level of deviance from prior practices) influence the IPC professionals' experience with facilitation activities during pandemics.

## Figures and Tables

**Table 1:** Representative studies of facilitation activities

Author(s), year and journal	Country	Research setting	Facilitation activities reported
Ritchie *et al.* (2020) , *Implementation Science and Communication*	USA	Primary mental care	Building relationships and creating a supportive environment Changing the system structure of care Planning and leading change efforts Transferring knowledge to support for on-going learning Assessing people and outcomes for program monitoring
Moussa *et al.* (2019) , *Journal of Change Management*	Not applicable	Not applicable	Goal setting Assessing progress and outcomes Providing tools and resources
Zipfel *et al.* (2019) , *BMC Health Services Research*	The Netherlands	Cardiac surgery	Intrinsic motivation for the change Speed of implementation Continuous evolution
Young *et al.* (2019) , *Journal of Health Organization and Management*	Australia	Diet and therapy clinics	Building relationships and trust Understanding the problem and stimulating change in data Negotiating and implementing the change Measuring, sharing, and reflecting on success
Harvey and Lynch (2017) , *Frontiers in Public Health*	Not applicable	Not applicable	Clarifying core elements of facilitation Recruiting and preparing to become facilitators Building the right networks to support and mentor facilitators
Lessard *et al.* (2016) , *Implementation Sciences*	Canada	Family medicine	Legitimization of change Formation of coalition Communication of project guidelines Empowerment of others to change Continuous improvement
Chreim *et al.* (2012) , *Journal of Health Organization and Management*	Canada	Especial medical service	Attention on power dynamics Persistent leadership Elimination of boundaries between groups Aligning incentives with desired practice changes
Dogherty *et al.* (2012) , *Implementation Sciences*	Canada	Tele practice, breast cancer care and distress management	Displaying and generating enthusiasm at the start of the project Thinking ahead in the process Taking on specific tasks, being available as needed Ensuring group remains on task and things are not missed

**Note(s):** Contemporary studies identified in a state-of-the-art literature review on facilitation activities in healthcare

**Table 2:** Descriptive information of interviews

No	Formal position	Dur. (min)
*Stage 1: Exploratory interviews*		
1	Head nurse of surgical center	69
2	Epidemiological surveillance coordinator	51
*Stage 2: Semi-structured interviews*		
3	IPC nurse	54
4	IPC coordinator	62
5	IPC nurse	55
6	IPC coordinator	49
7	IPC nurse	65
8	IPC nurse	58
9	IPC nurse	72
10	Infectious disease physician	57
11	IPC coordinator	68
12	IPC nurse	52
13	IPC coordinator	73
14	IPC nurse	68
15	IPC nurse	41
16	IPC consultant nurse	47
17	Infectious disease physician	55
18	IPC nurse	42
19	IPC nurse	71
20	IPC nurse	68
21	IPC nurse	58
22	Infectious disease physician	40
23	IPC consultant nurse	51
*Stage 3: Follow-up interviews*		
24	IPC coordinator	55
25	Infectious disease physician	72
